# Late HBsAg seroreversion of mutated hepatitis B virus after bone marrow transplantation

**DOI:** 10.1186/1471-2334-13-223

**Published:** 2013-05-16

**Authors:** Axel Schubert, Detlef Michel, Thomas Mertens

**Affiliations:** 1 Institute for Virology, Ulm University Medical Center, Albert-Einstein-Allee 11, Ulm, 89081, Germany

**Keywords:** Hepatitis B virus, Seroreversion, Reactivation, Immune escape mutation, HSCT, Immunosuppression

## Abstract

**Background:**

About ninety percent of immunocompetent adults recover from hepatitis B virus (HBV) infection within 6 months after transmission. The infection is considered to be terminated if the antibodies (HBsAb) to the hepatitis B surface antigen (HBsAg) become detectable and the HBsAg and Hepatitis B virus DNA (HBV DNA,) are no longer perceptible. After recovery from an acute infection, the detection of HBsAb is assumed to indicate lifelong immunity. However, after initiation of severe immunosuppression, HBV reactivation, as detected by HBsAg seroreversion may be observed in patients with previously resolved HBV infections.

**Case presentation:**

We present an unusual case of a 64-year-old Caucasian woman showing clinically apparent HBV seroreversion more than 45 months after hematopoietic stem cell transplantation (HSCT). Despite living without immunosuppressive agents for more than 40 months, she developed a fulminant HBV infection with detection of a mutated hepatitis B virus carrying two immune escape mutations (D144E/G145R) in the HBsAg (HBsIE mutation).

**Conclusion:**

After HSCT, the absence of risk factors such as strong immunosuppression and graft-versus-host disease decreases the risk of HBV seroreversion but may rearward seroreversion to a later time. Therefore, when monitoring HSCT, patients with serological markers of a resolved HBV infection [HBcAb + (hepatitis B core antibody), HBsAb+, and HBsAg−], the follow up has to be extended over several years to exclude HBV reactivation with HBsAg seroreversion. Furthermore, this case demonstrates the complexity of virus evolution after HBsAg seroreversion as a result of immunosuppression after HSCT.

## Background

Due to the low percentage of patients with prior hepatitis B virus (HBV) infections, HBsAg seroreversion is a rare complication after hematopoietic stem cell transplantations (HSCT) in Germany. But this complication may turn out to be an increasing problem in future, since HSCT is becoming a standard treatment even in elderly patients (>60 years) who are expectably more frequently positive for serum markers of a resolved HBV infection (HBcAb +, HBsAb +, and HBsAg -) [1].

Previous studies in HSCT patients report HBV seroreversion in patients with serologically resolved HBV infections mainly within the first 12–24 months after the initiation of severe immunosuppression [2-6]. During HBV reactivation of a prior resolved HBV infection, HBAg seroreversion usually leads to the loss of HBsAb and reappearance of HBsAg. In some cases even a complete loss of all antibodies against HBV antigens may be observed over years despite a highly viremic infection. In these cases immunological tolerance against HBV antigens has been suggested [7]. During immunosuppression, HBV infection remains clinically silent but may lead to acute liver failure during T cell restoration. In most of these reported cases, prophylaxis with antiviral agents was not performed.

HBV seroreversion is assumed to be due to “reawakened” covalently closed circular HBV DNA (cccDNA) which can persist (especially in the hepatocytes) for a considerable time, even after having terminated clinically and serologically the HBV infection [8-10]. Furthermore, reinfection of hepatocytes by HBV after reactivation in other latently infected cells [*e.g.* peripheral blood mononuclear cells (PBMCs)] has been previously discussed [11,12].

Although HBsAg seroreversion was first reported in the early 1990s [13], the risk for HBsAg seroreversion in patients with resolved HBV infection undergoing immunosuppression remains controversial in literature. The incidence ranges from <10% [14] to around 20% [15-19] up to approximately 90% [3].

Reasons for differences in the reported rates of HBsAg seroreversion are heterogenicity of the immunosupressed patients at risk and the fact that the term HBV reactivation has not been used always distinctly. In some reports there is no clear differentiation between HBV reactivation from active or chronic HBV infection (called flares) and HBV reactivation caused by HBsAg seroreversion. This may also be true for so called occult HBV infection where diagnosis depends mainly on the sensitivity of the HBsAg and in particular the HBV DNA assay [6,20]. In several studies using comparable definitions the following main risk factors for seroreversion have been discussed, which all take lastly influence on duration and severity of immunosuppression. Different immunosuppressive drugs and treatment regimens, different underlaying onco-hematological diseases before HSCT or GvHD occurrence after HSCT may influence HBsAg seroreversion. It is obvious from many studies that the rates of seroreversions significantly increase with time after HSCT with ongoing immunosuppression [4,6,15-19]. Donor immunity against HBV and the amount of HBsAb in the recipient are often claimed to be relevant for the probability of early HBsAg seroreversion [3,14]. Patients who show isolated HBcAb positivity seem to carry a greater risk for early HBsAg seroreversion after HSCT, too [17].

## Case presentation

A 64-year old Caucasian woman had undergone a bone marrow transplantation in September 2006 after diagnosis of acute monocytic leukemia (AML). The patient received induction therapy on March 1, 2006, and at this point all the markers of a previously resolved HBV infection (HBcAb +, HBsAb +, HBsAg −) were observed. In September 2006, following complete remission after rituximab, busulfan, and fludarabine therapy, the patient was treated by allogeneic blood stem cell transplantation.

Except for a short steroid therapy in February 2007 to treat an immune thrombocytopenic purpura, the patient received no further immunosuppressive drugs. In May 2009 (33 months later), all the serological markers characteristic of a resolved HBV infection were still observed in the patient; furthermore, no HBV DNA could be detected [detection limit approximately 100 genome equivalents per milliliter (ge/ml)].

**Figure 1:**
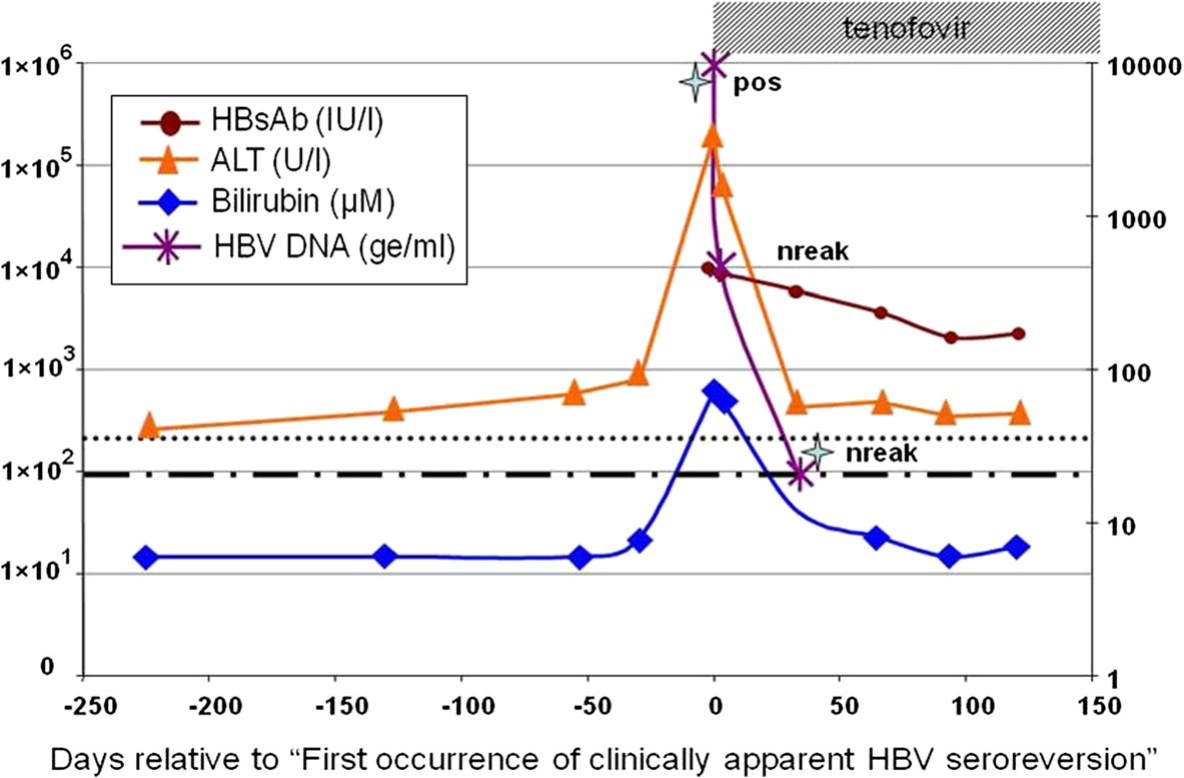
**HBV serology and clinical pathology.** Alanine transaminase (ALT) concentrations (U/l) are indicated by triangles (orange triangle) and bilirubin values by squares (blue square). Limit of “normal blood values” are shown by dashes (∙∙∙∙) ALT >35 U/L;(----) Billirubin >(21 μM). HBsAb values are indicated by circles (red circle). Detection of HBsAg is indicated by “pos” (HBs-Antigen detected) or “nreak” (HBsAg negative by COBAS-HBsAg II). Quantitative detection of HBV DNA is indicated by small asterisks (violet asterisks) and sample time points used for HBV genotyping by stars (skyblue stars). Duration of tenofovir therapy: dashed box.

In June 2010, she complained of abdominal pain which had lasted for one week and was admitted to the hospital. Initial laboratory investigations revealed elevated levels of aminotransferase (3295 U/l) and bilirubin (73 μM) (Figure 1).

Despite being still positive for HBsAb (431 IU/l, COBAS-HBsAg II assay, Roche) in serum 46 months after HSCT, tenofovir therapy (300 mg/day) was initiated in view of a low positive HBsAg. The next serum sample taken 3 days later resulted negative for HBsAg and all markers associated with a resolved HBV infection were observed. Both sera gave low positive HBsAg results when tested later by the HBsAg Qualitative [ABBOTT (Architect)] assay. All further sera remained negative in both assays.

However, HBV reactivation was clearly detected by in-house qPCR, since both samples (days 0 and 3 after initiation of tenofovir therapy) showed significant levels of HBV DNA (5 ×10^8^ ge/ml and 1 × 10^4^ ge/ml, respectively). One month later during the course of therapy, the HBV DNA level had dropped to 200 ge/ml and another month later, HBV DNA was undetectable and remained undetectable till date.

Given the rare occurrence of both HBsAb and HBsAg being simultaneously positive, the samples from day 0 and one month after the initiation of tenofovir therapy were tested for the presence of an immune escape mutation in the HBsAg (HBsIE mutation). Surprisingly, in both samples (genotype D) two identical mutations (D144E and G145R, described in literature as HBsIE mutations) were identified.

## Discussion

Frequently discussed risk factors for developing HBV reactivation by seroreversion are low pretransplant HBsAb levels, donor immunity, appearance of GVHD and severity and duration of immunosuppression. In this case, immunosuppression was relatively short and GVHD was not observed, but rituximab (R-CHOP) had been administered. Unfortunately, no quantitative determination of HBsAb before immunosuppression was performed. The stem cell donor of our patient was a non responder (HBsAb negative) after prior HBV vaccination. Case reports demonstrating HBV seroreversion after more than 30 months since initiation of immunosuppression are very rare [3,21]. In those studies, the time point of HBV seroreversion depended mainly on the HBsAb concentration before initiation of immunosuppression. Given the fact that the patient’s serum was still HBsAb positive more than 2.5 years after HSCT, high antibody titers prior to the initiation of induction therapy can be assumed. All of these factors may have led to the late occurrence of seroreversion. The simultaneous detection of HBsAb and HBsAg led to the discovery of two HBsIE mutations explaining the lower HBsAg sensitivity of the two commercial platforms used in this study (Abbott and Roche) [22]. The numbers of HBsIE mutations described under such circumstances are very low, and to our knowledge, have been mentioned in only one other case report. Knöll et al. (2007) reported two cases of HBV seroreversion after HSCT where HBsIE mutations were detected [3]. Interestingly, in both cases HBsIE double mutations were also identified (D144G/G145E and P142L/G145R). The presence of an uncommon double mutation (D144E/G145R) in HBsAg may indicate HBV reinfection from PBMCs because the mutation G145R appears to occur more often in PBMCs [23]. The D144E mutation may have been acquired during replication under immune selective pressure. Despite the high level of HBV DNA and the appearance of the HBsIE mutation, the HBV DNA level was reduced to below the detectable limit after 2 months of tenofovir therapy. Therefore, tenofovir appears to be an effective treatment in HBV infection after HBsAg seroreversion.

## Conclusions

The occurrence of a clinically apparent HBsAg seroreversion more than 50 months after HSCT and initiation of immunosuppression shows that HBV reactivation has to be monitored for more than 4 years after HSCT. The absence of main risk factors like GVHD and marked immunosuppression as well as the long lasting presence of HBsAb decreases the risk of a seroreversion, but may postpone HBV seroreversion to later times.

We could show that the absence of HBsAg with simultaneous detection of HBsAb does not completely exclude HBV reactivation after immunosuppressive therapy. Therfore monitoring should include HBsAb, HBsAg, and HBV DNA besides alanine aminotransferaseas. No evidence based recommendations for prophylaxis with antiviral agents can be given now, especially concerning duration of the therapy. The premature ending of therapy may result in postponed HBsAg seroreversion and unrecognized seroreversion under therapy may result in selection of drug resistance. Finally, prophylaxis with passive (HBsAb) or even active (HBsAg) immunization may induce immune escape mutations in the YMDD region and may simultaneously lead to the emergence of drug resistance even in previously untreated patients.

## Consent and ethical background

Written informed consent was obtained from the patient for publication of this case report. A copy of the written consent is available for review by the Editor-in-Chief of this journal.

## Competing interests

The authors declare that they have no competing interests.

## Authors’ contributions

AS was involved in data analysis and researched the background literature on the case and wrote the first draft. DM contributed to discussions, analysis of the case and was involved data analysis. TM provided expert opinion on analyzing the case and participated in writing the manuscript. All authors read and approved the final manuscript.

## Authors’ information

MT is professor and director of the institute of Virology, University Medical Center Ulm, Germany. SA and MD are laboratory managers at the same institute.

## Pre-publication history

The pre-publication history for this paper can be accessed here:

http://www.biomedcentral.com/1471-2334/13/223/prepub
